# Stretching the Equilibrium Limit of Sn in Ge_1–*x*_Sn_*x*_ Nanowires: Implications for Field Effect Transistors

**DOI:** 10.1021/acsanm.0c02569

**Published:** 2021-02-03

**Authors:** Subhajit Biswas, Jessica Doherty, Emmanuele Galluccio, Hugh G. Manning, Michele Conroy, Ray Duffy, Ursel Bangert, John J. Boland, Justin D. Holmes

**Affiliations:** † School of Chemistry and Advanced Materials and Bioengineering Research (AMBER) Centre, University College Cork, Cork T12 YN60, Ireland; ‡ Tyndall National Institute, University College Cork, Cork T12 R5CP, Ireland; § School of Chemistry and AMBER, Trinity College Dublin, Dublin 2, Ireland; ∥ TEMUL, Department of Physics, Bernal Institute, University of Limerick, Limerick V94 T9PX, Ireland

**Keywords:** germanium−tin, supercritical fluid, nonequilibrium alloy, bottom-up growth, field-effect transistor

## Abstract

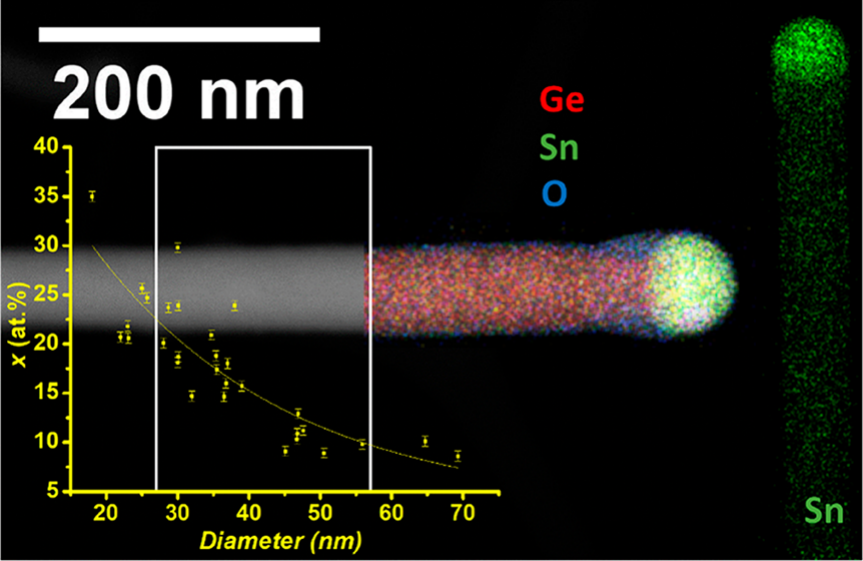

Ge_1–*x*_Sn_*x*_ nanowires incorporating a large amount of Sn would be useful for mobility enhancement in nanoelectronic devices, a definitive transition to a direct bandgap for application in optoelectronic devices and to increase the efficiency of the GeSn-based photonic devices. Here we report the catalytic bottom-up fabrication of Ge_1–*x*_Sn_*x*_ nanowires with very high Sn incorporation (*x* > 0.3). These nanowires are grown in supercritical toluene under high pressure (21 MPa). The introduction of high pressure in the vapor–liquid–solid (VLS) like growth regime resulted in a substantial increase of Sn incorporation in the nanowires, with a Sn content ranging between 10 and 35 atom %. The incorporation of Sn in the nanowires was found to be inversely related to nanowire diameter; a high Sn content of 35 atom % was achieved in very thin Ge_1–*x*_Sn_*x*_ nanowires with diameters close to 20 nm. Sn was found to be homogeneously distributed throughout the body of the nanowires, without apparent clustering or segregation. The large inclusion of Sn in the nanowires could be attributed to the nanowire growth kinetics and small nanowire diameters, resulting in increased solubility of Sn in Ge at the metastable liquid–solid interface under high pressure. Electrical investigation of the Ge_1–*x*_Sn_*x*_ (*x* = 0.10) nanowires synthesized by the supercritical fluid approach revealed their potential in nanoelectronics and sensor-based applications.

## Introduction

Alloying group IV semiconductors, such as Ge or Si with group IV metals such as Sn, can lead to a direct bandgap semiconductor.^1−5^ Theoretically, increasing the amount of Sn in bulk Ge results in a direct bandgap at a Sn concentration between 6.5 and 25 atom %, an inverse semimetallic bandgap when Sn is >25 atom %, and an inverse spin–orbit split-off at a Sn content between 45 and 85 atom %.^6^ Although a direct bandgap can be achieved in Ge_1–*x*_Sn_*x*_ alloy for Sn content as low as *x* = 0.06, a certain degree of Γ–L mixing is observed for Sn contents in the region 0.06 < *x* < 0.1.^6,7^ The drive to incorporate high Sn concentrations (*x* > 0.1) in Ge_1–*x*_Sn_*x*_ alloy nanowires can be partly attributed to the presence of this band-mixing at lower Sn content Ge_1–*x*_Sn_*x*_ alloys.^7−11^ Recent theoretical calculations have also supported that the indirect-gap to direct-gap transition proceeds via the continuous transition–with increasing *x*.^11,12^ The optical and optoelectronic properties of an alloy can be influenced by the alloy composition, as demonstrated both theoretically^13^ and experimentally.^14−16^ A definitive transition to a direct bandgap is required, with large enough Sn incorporation, for the use of Ge_1–*x*_Sn_*x*_ in efficient optoelectronic devices, such as photodiodes and photodetectors and photonic devices without the need for any external force such as induced strain.^17,18^

Additionally, in the case of Ge_1–*x*_Sn_*x*_, the larger incorporation of Sn into Ge (*x* = 0.15–0.3) can increase the energy difference between L and Γ valleys, which can shift the emitted wavelengths toward the mid-infrared (>3 μm).^19,20^ This improves the efficiency of the GeSn-based light sources in terms of lasing threshold and operating temperature and makes them applicable for fully integrated Si optoelectronic and photonic systems used in mid- and far-infrared applications. Significantly, GeSn nanowires with high Sn incorporation, i.e., 19 atom %, have also demonstrated high electrical conductivity values and semiconductor behavior.^21^ Mobility enhancement for both electrons and holes is predicted for GeSn alloy field effect transistor (FET) with a high Sn content due to deformation potential acoustic (dp-ac) phonon scattering, as well as alloy scattering.^22^ However, there have been very few reports on synthesizing Ge_1–*x*_Sn_*x*_ nanowires with *x* > 0.1.

Recently, the growth of GeSn nanowires has been achieved by both top-down^23^ and bottom-up approaches,^8,24,25^ including the bottom-up growth of Ge/GeSn core/shell nanowires.^26−29^ Bottom-up growth of GeSn nanowires was mainly achieved by using in situ formed Sn as a catalyst or using third party catalysts such as Au, AuAg, AuSn, etc.^30,31^ Growth methods and critical growth constraints for the growth of Ge_1–*x*_Sn_*x*_ nanowires are detailed in a couple of recent reviews.^32,33^ However, in most of these growth methods, Sn incorporation in the alloy nanowire is limited to below 10 atom %. In a significant development, Seifner et al. reported the synthesis of high Sn content Ge_1–*x*_Sn_*x*_ nanowires (*x* ≈ 0.19) via a chemical vapor deposition approach.^34^ However, the effort to integrate larger Sn (>25 atom %) in one-dimensional (1D) nanostructures via solution phase approach resulted in short and thick nanorod structures.^35^ The incorporation of Sn in Ge at >30 atom % has mostly been reported in nanoparticles.^36^

Recent reports from our group have demonstrated highly crystalline Ge_1–*x*_Sn_*x*_ nanowires with a Sn content of ≈9 atom % via metal catalyzed vapor–liquid–solid (VLS) growth methods. The nonequilibrium incorporation (much greater than ≈1 atom % equilibrium solubility of Sn in Ge) of Sn in the Ge lattice is attributed to “solute trapping”.^8,37^ However, to the best of our knowledge, reports on “solute trapping” of impurities in the host lattice generally consider isobaric conditions and the effect of pressure has not been explored and described.^38,39^ “Solute trapping” of impurities during the VLS growth of nanowires is likely to be influenced by pressure. This could be due to a change in the metastable solubility of Sn at the liquid–solid growth interface. Additionally, pressure would be expected to influence the growth kinetics of Ge_1–*x*_Sn_*x*_ nanowires.^40,41^ Rapid decomposition of precursor under high pressure plays a significant role in altering the nanowire growth kinetics, as the growth kinetics may no longer be dominated by the crystallization rate at the liquid/solid interface between catalyst and nanowire but rather have a nontrivial contribution from the diffusion and incorporation of the growth species into the nanowire catalyst (i.e., the vapor/liquid interface) and at the triple phase interface.^41^ This altered growth kinetics could influence impurity incorporation via kinetic dependent solute trapping.^42^ Thus, introduction of pressure as an additional parameter in a VLS-like nanowire growth could positively influence the impurity (e.g., Sn) incorporation in an alloy nanostructure.

In this article, we report the ability to increase the concentration of Sn in Ge nanowires beyond 25 atom % by introducing high pressure as a growth constraint. We have utilized a supercritical solvent medium for the Ge_1–*x*_Sn_*x*_ nanowire growth. High pressures (∼21 MPa) result in Ge_1–*x*_Sn_*x*_ nanowire growth with 0.1 ≤ *x* ≤ 0.35, much higher than previously reported for Sn incorporation in Ge 1D lattices. Also, given the scarcity of reports detailing the electronic characterization of bottom-up grown Ge_1–*x*_Sn_*x*_ nanowires in (FET)-like devices,^21^ we report the most important FET electronic figures-of-merit for nominally undoped Ge_1–*x*_Sn_*x*_ nanowires with 10 atom % Sn incorporation.

## Results and Discussion

**Figure 1:**
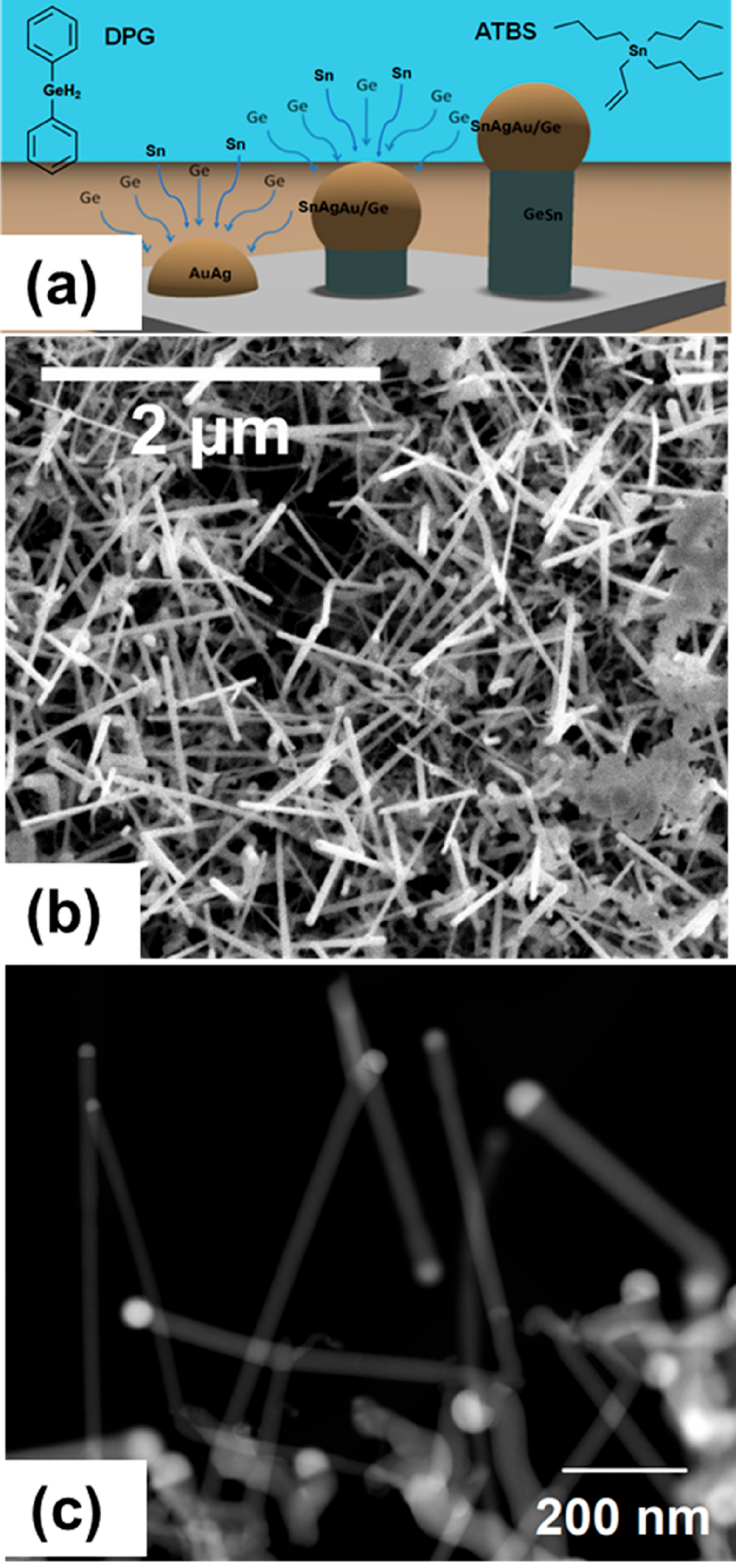
(a) Growth scheme of the Ge_1–*x*_Sn_*x*_ nanowires via VLS paradigm. (b) SEM image of Ge_1–*x*_Sn_*x*_ nanowires highlights the uniformity of the nanowires across the substrate. The presence of Sn agglomerates and the tendency of the Ge_1–*x*_Sn_*x*_ nanowires to cluster can be seen in (b). (c) STEM image displays minimal tapering and the presence of growth seeds.

A very high Sn content could impact on the crystal quality and stability of the alloy, along with the optical and electronic properties. Thus, it is interesting to look into the less explored region of Sn incorporation, i.e., beyond 25 atom % Sn, in Ge_1–*x*_Sn_*x*_ alloy nanowires. To overcome the limitations of Sn incorporation (≈10 atom % Sn)^8,25,37^ in Ge_1–*x*_Sn_*x*_ nanowires via atmospheric pressure chemical vapor deposition (CVD) growth, pressure was introduced as an additional growth parameter in a supercritical fluid (SCF) environment. A supercritical-fluid–liquid–solid (SFLS) approach, using toluene as the SCF phase and Au_0.90_Ag_0.10_ alloy nanoparticles as growth catalysts, was deployed for growing Ge_1–*x*_Sn_*x*_ nanowires on the surface of Si (001) substrates at a growth temperature of 405 °C and a pressure of 21 MPa (see Supporting Information for detailed experimental procedure). Diphenyl germane (DPG) and allyltributylstannane (ATBS) precursors were used as sources of Ge and Sn in the reaction, respectively. A vapor–liquid–solid (VLS) growth is liable for the bottom-up fabrication of Ge_1–*x*_Sn_*x*_ nanowires. A schematic in Figure 1a shows the general growth method of Ge_1–*x*_Sn_*x*_ nanowires. The high pressure can encourage vapor phase decomposition of the growth precursors which influence the nanowire growth kinetics.^39^ A kinetic dependent solute trapping mechanism is believed to be liable for Sn incorporation in Ge_1–*x*_Sn_*x*_ nanowires, where nanowires with faster growth rates lead to higher Sn incorporation in the nanowires.^8,37^

Scanning electron microscopy (SEM) and scanning transmission electron microscope (STEM) images in Figure 1 show the growth of Ge_1–*x*_Sn_*x*_ nanowires in a supercritical toluene environment at a pressure of 21 MPa and at a temperature of 405 °C. Au_0.90_Ag_0.10_ seeds sparsely deposited on a Si substrate resulted in the formation of clusters of Ge_1–*x*_Sn_*x*_ nanowires (Figure 1b). The formation of Sn aggregates in the sample can also be seen in the SEM image of Figure 1b; clusters of Sn were observed across the entire substrate. This Sn segregation was not apparent in samples of Ge_1–*x*_Sn_*x*_ nanowires grown by atmospheric pressure CVD.^8,37^ The diameters of the nanowires synthesized were between 20 to 70 nm, with a mean diameter of 34.2 nm (standard deviation of 14.2 nm) and with lengths of ≤2 μm (Figure 1). The observed diameter range (diameter distribution of nanowires is given in Figure S1a in Supporting Information) and the mean diameter is also different compared to the atmospheric CVD grown nanowires with the same starting catalysts (Au_0.90_Ag_0.10_ nanoparticles) and Sn precursors (ATBS). The Sn segregation in the sample and the wider diameter range of nanowires, compared to the CVD grown nanowires, are likely attributable to the highly reactive nature of the gas phase reaction involving the Sn precursor (ATBS) in the SCF atmosphere under high pressure.^43^

A closer look at the nanowires by STEM (Figure 1c) provided further proof of the uniform morphology of the nanowires. Tapering of the nanowire was negligible. Growth at higher temperature, with different precursors (e.g., tetraethyltin) and using Au nanoparticle catalyst, resulted in a lower nanowire yield (compared to the spherical aggregates in the sample), undesirable nanowire morphology (e.g., short, tapered nanowires), and lower Sn incorporation (average Sn incorporation of <10 atom %) in the Ge_1–*x*_Sn_*x*_ nanowires (see EDX and SEM analysis in Figure S1 in the Supporting Information). The aforementioned growth condition of 405 °C growth temperature, Au_0.90_Ag_0.10_ alloy nanoparticle catalysts, and ATBS as the Sn precursor were ideal to obtain the best quality GeSn nanowires with high Sn incorporation in the SCF growth setup. The hemispherical catalyst seeds seen at the tips of the nanowires (visible in Figure 1c) verify a catalytic VLS-like growth mechanism. However, unlike the CVD-grown Ge_1–*x*_Sn_*x*_ nanowires previously reported,^8,37^ the nanowires grown using this SCF approach did not appear to contain a bulb of Ge_1–*x*_Sn_*x*_ (*x* ≈ 0.5) surrounding the catalytic seeds.

**Figure 2:**
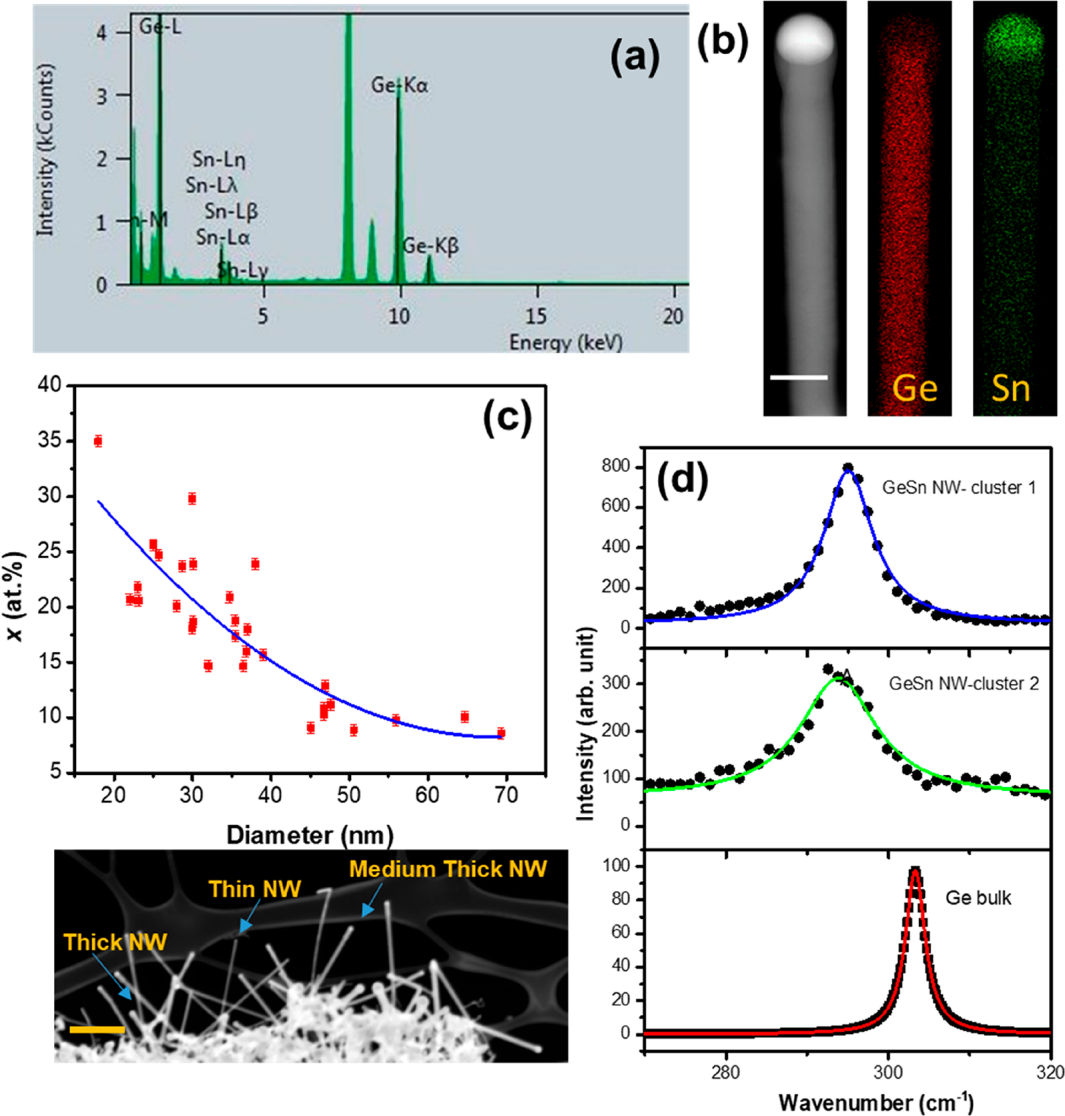
EDX elemental analysis of high Sn content Ge_1–*x*_Sn_*x*_ nanowires. The EDX spectrum in (a) is representative of these nanowires with *x* > 0.1. (b) Elemental mapping of a Ge_1–*x*_Sn_*x*_ (*x* = 0.18) nanowire shows the homogeneous Sn distribution in the body of the nanowire and a Sn rich tip. Ge is denoted by red and Sn by green. Scale bar denotes 50 nm. (c) Sn incorporation vs diameter plot shows a dramatic change in the Sn composition of the nanowire with the small variation in nanowire diameter (the blue line is guide for the eyes). *Y*-axis error bar denotes standard error of 0.5 atom % in the EDX quantification. The STEM image attached below the plot shows the relation between diameter and nanowire length (scale bar denotes 200 nm). (d) Raman spectra from a GeSn nanowire cluster shows large Raman shift compared to Ge bulk.

Energy dispersive X-ray (EDX) point analysis on the Ge_1–*x*_Sn_*x*_ nanowires revealed a mean Sn composition of ∼17.1 atom % . Measurements from 50 nanowires were considered to calculate mean concentration, and a representative elemental spectrum is shown in Figure 2a. EDX analysis also revealed no elemental signal associated with Au or Ag, suggesting either Au or Ag present in the nanowire is below the EDX detection limit (0.5 atom %) or Au or Ag was not incorporated into the nanowires from the catalyst. This is an important factor when considering the implementation of nanowires in optoelectronic and nanoelectronic devices, as Au can act as a deep trap and a very low content of Au could act negatively for electronic transport. Sn incorporation in the SCF grown Ge_1–*x*_Sn_*x*_ nanowires was found to vary significantly from one nanowire to another, i.e., ∼10–35 atom % (representative EDX point analysis spectra for nanowires with different Sn contents is given in Figure 2a and Figure S2 in the Supporting Information). Notably, the incorporation of Sn in Ge at concentrations of >10 atom % is well above the equilibrium solubility (1 atom %) limit. Therefore, to ensure that Sn was homogeneously distributed throughout the body in the Ge_1–*x*_Sn_*x*_ nanowires, i.e., no Sn segregation in the bulk or surface of the nanowires or a gradual decrease in the Sn content from the seed to the end of a nanowire, EDX elemental maps were obtained for individual nanowires. Figure 2b displays the individual elemental maps for Ge and Sn (Sn denoted by green, Ge by red) and corresponding dark-field STEM image for a representative nanowire with 18 atom % Sn incorporation. EDX line scan of Ge_1–*x*_Sn_*x*_ nanowires with higher a Sn content (*x* > 0.25) is also shown in the Supporting Information (Figure S3a). The lack of Sn segregation or clustering is verified by the absence of bright green spots (corresponding to Sn) in the elemental map in Figure 2b. The formation of a Sn rich seed is also clearly visible from the elemental map, also reported for CVD grown Ge_1–*x*_Sn_*x*_ nanowires grown at a similar tempreature.^8,37^ However, the spherical catalytic particles at the tip of the nanowires contain less Sn (∼75 atom % compared to >90 atom %; see Figure S4 in the Supporting Information) in the resulting AuAgSn alloys compared to the CVD grown GeSn nanowires grown with same AuAg nanoparticle catalysts and Sn precursor but grown at a higher temperature (440 °C). Transformation of the initial AuAg alloy seeds to Sn rich seeds at the tips of the nanowires after growth is due to the infinite solubility of Sn in Au_0.90_Ag_0.10_ at our growth temperature of 405 °C. The nanowires grown in the SCF solvent depicted a Sn rich catalytic seed, similar to the CVD grown GeSn nanowires. An oxide rich layer can also be observed in dark-field STEM image in Figure 2b, close to the nanowire seed. To ensure that there was no influence from this potentially Sn rich oxide layer on the calculated Sn composition of the Ge_1–*x*_Sn_*x*_ nanowires, EDX point analysis was conducted at a distance of >100 nm from the seed–nanowire interface.

Despite the relatively narrow diameter range, mean diameter of around 34 nm, the Ge_1–*x*_Sn_*x*_ nanowires tended to remain clustered together, making direct comparisons between nanowire lengths challenging. The dark field STEM image in Figure 2c clearly shows the diameters of Ge_1–*x*_Sn_*x*_ nanowires between a lower (∼20 nm) and higher (∼70 nm) range. However, it is apparent from the STEM image in Figure 2c that small diameter nanowires were longer than their larger diameter counterparts (Figure 2c and length–diameter plot in Figure S5 in the Supporting Information). This is contrary to diameter dependent lengths observed for supersaturation-limited VLS growth of Ge nanowire by CVD,^40^ where the Gibbs–Thomson effect is liable for faster growth kinetics in larger diameter nanowires. This discrepancy may be due to the higher reactivity of the precursors in an SCF growth regime under high pressure compared to CVD growth. The pressure component in the SCF based VLS-like growth may promote the kinetics associated with the absorption of growth species at a catalyst surface. In comparison, in a crystallization limited process, observed in CVD growth of Ge nanowires, the growth rate is influenced by the nucleation and crystal growth at a liquid/solid interface. In SCF assisted high-pressure growth, faster precursor decomposition results in a large chemical potential in the vapor (SCF in our case) phase. As a result, both the crystallization at the liquid–solid interface and the incorporation of growth material into the catalyst influence the final growth kinetics of nanowires. Hence, the diameter (*d*) dependence on the growth rate (*v*) does not follow the conventional Gibbs–Thomson size effect but instead can be represented by a diffustion-limited growth model given by , where *v*_0_ is the growth rate of the nanowire at infinite diameter (*d*), Ω^S^ is the molar volume in the solid phase, and σ^S^ is the surface tension of the nanowires.^41^ Hence, when *d* is infinite, the growth rate is restricted to *v*_0_, but when *d* is minimal, the growth rate increases. This model justifies the inverse relationship between the nanowire diameter and growth kinetics (Figure 2c and Figure S5 in the Supporting Information) for Ge_1–*x*_Sn_*x*_ nanowires grown via the SCF approach, with smaller diameter nanowires having longer lengths than larger diameter nanowires.

Sn is usually incorporated in the Ge nanowire lattice in a VLS-like growth via the solute trapping mechanism, a kinetically driven process.^37^ Solute trapping describes the incorporation of impurities by solute redistribution at the catalyst–nanowire interface. At the catalyst–nanowire (liquid–solid) interface, the difference in atomic concentration in the different phases can influence the trapping of impurity adatoms on the high energy sites of the crystal lattice. Further insights into the solute trapping of Sn in Ge is given in our previous papers on the CVD-grown Ge_1–*x*_Sn_*x*_ nanowires.^8,37^ The large difference in the Sn concentration between the liquid eutectic seed (Figure S4 in the Supporting Information shows large Sn content in the catalytic tip) and nanowire can result in the solute trapping of Sn by each succeeding layer of the nanowire, assuming a layer-by-layer nanowire growth. Additionally, Sn can also be adsorbed on the nanowire side facets, which can further diffuse via the nanowire bulk or surface. The bulk diffusion of Sn through the nanowire sidewall should not be prominent for the SCF grown GeSn nanowires due to the very low solid solubility and diffusivity of Sn in Ge. However, small inclusion of Sn in the GeSn nanowires via surface diffusion and inclusion through the triple phase interface is possible. But the incorporation of Sn through this pathway could be hindered by Sn’s negligible diffusion in Ge at the growth conditions, the elastic strain at the seed–nanowire interface due to epitaxial mismatch between Ge and Sn, and the lack of the presence of truncating side facets at the seed–nanowire interface (which can act as attractive sites for Sn aggregation). However, small inclusion of Sn via surface diffusion and inclusion through the triple phase interface cannot be ruled out. Although we did not observe any tapering or peaks the EDX line scan associated with Sn near the surface region (Figure S3b in the Supporting Information), a very thin adsorbed layer of Sn on the nanowire surface is visible in the EDX map of the nanowire (Figure S3c in the Supporting Information).

In a kinetics dependent “solute trapping” model,^39^ faster growth rates lead to greater impurity incorporation; thus Ge_1–*x*_Sn_*x*_ nanowires with smaller diameters and faster growth kinetics should display a high Sn content. The high Sn contents (∼25–35 atom %) were observed (Figure 2c) for the smallest diameters (∼20–30 nm) nanowires, whereas nanowires with larger diameters (>50 nm) contained 9–10 atom % Sn. Of note, as the diameters of most of the nanowires are between 30 and 40 nm (diameter distribution of nanowires is given in the Supporting Information, Figure S1), a large number of the Ge_1–*x*_Sn_*x*_ nanowires have Sn content between 15 and 20 atom %. This large discrepancy of Sn inclusion in nanowires of different diameter has not been previously observed for the CVD grown Ge_1–*x*_Sn_*x*_ nanowires, although fluctuation in the Sn inclusion along the length of tapered nanowire and different Sn incorporation in different segments of GeSn branched nanowires were previously observed.^24,44^ Additionally, the impact of growth kinetics on Sn incorporation in CVD grown Ge_1–*x*_Sn_*x*_ nanowires has been reported.^37^ However, the enormous inclusion of Sn in small diameter nanowires under SCF conditions cannot be explained solely by nanowire growth kinetics. A 10-fold change in the solid solubility was observed for a Si–Al system under high pressure.^45^ Though the pressure applied to change the solid solubility of Si in Al was much larger (almost 100 times) than the pressure applied (21 MPa) in our nanowire growth experiment, high pressure can contribute toward the enhancement of solubility of Sn impurities in the liquid eutectic alloy. A high level of Sn inclusion in the nanowire could be the result of an increased metastable solubility of Sn at the catalyst–nanowire interface, under high pressure and for smaller nanowire diameters. The effect of pressure in the solid solubility of Sn in Ge and eutectic solubility of Sn in the eutectic alloy needs further verification as no such observation on the pressure effect on the Sn–Ge phase diagram is reported in the literature.

Impurity adatoms can be trapped on the high energy sites of the crystal lattice at a high solidification rate which can lead to the formation of metastable solids (Ge_1–*x*_Sn_*x*_ with *x* > 0.01) at the growth front (catalyst–nanowire interface).^37^ This deviation of the chemical equilibrium at the interface is influenced by the interfacial diffusion speed, *V*_DI_, a kinetic parameter where . *V*_DI_ is a ratio of the diffusion coefficient at the interface (*D*_I_) and the characteristic distance for the diffusion jump (λ) which is equal to the width of the solid–liquid interface, i.e., equivalent to the diameter of the nanowire.^39^ Solute trapping generally increases with high interfacial diffusion speed, interface velocity, and low bulk diffusion speed. Thus, approaching a lower diameter regime for high interfacial diffusion and interface velocity could be beneficial for larger Sn incorporation. It is to be noted that the very high Sn content in Ge nanowires is only observed for nanowires with diameter less than 30 nm (Figure 2c), whereas the Ge_1–*x*_Sn_*x*_ nanowires of diameter >50 nm depicted Sn incorporation in the order of 9–10 atom %, similar to that of CVD grown nanowires. Additionally, another parameter, equilibrium partition coefficient, also affects the trapping of impurities.^37,39^ The equilibrium partition coefficient is characterized by the difference in atomic concentration in the different phases. For the high-pressure grown Ge_1–*x*_Sn_*x*_ nanowire, higher decomposition of the precursors could result in a larger difference in the atomic concentration between phases and hence an altered equilibrium partition coefficient and solute trapping. Further understating of the role of the pressure on the solute trapping of Sn in Ge is delegated to a future study.

Raman scattering is an effective tool for estimating the structural and chemical environment in the core of a nanowire. Raman measurements were performed on individual Ge_1–*x*_Sn_*x*_ nanowire clusters at a very low laser power to avoid laser-induced heating. In bulk Ge (Ge wafer, Umicore), the Ge–Ge longitudinal optical (LO) vibration is observed at 303.3 cm^–1^, due to the triply degenerated E_2g_ vibration (Ge–Ge mode). In Ge_1–*x*_Sn_*x*_ alloys the Ge–Ge mode moves toward a lower frequency of 295.0 and 293.8 cm^–1^ for two different clusters of nanowires (Figure 2d) and shows asymmetry in the lower energy side of the spectrum due to the development of a Ge–Sn coupled vibrational mode with a high Sn concentration. A red shift of 8.3 and 9.5 cm^–1^ of the Ge–Ge LO mode was observed for the Ge_1–*x*_Sn_*x*_ nanowire clusters with very high Sn incorporation, from bulk Ge. This Raman shift is of a similar order to the large Raman shift, of the order of 6–10 cm^–1^, observed for strain-free Ge_1–*x*_Sn_*x*_ thick films with 12–15 atom % Sn incorporation.^46^ As the participation of compressive and tensile strain toward the Raman shift is not justified for nanowire samples, due to the large surface area of the nanowires, the total shift of Ge–Ge frequency to lower values for the grown Ge_1–*x*_Sn_*x*_ nanowires is mainly attributed to Sn incorporation in the Ge lattice and alloy disorder imposed by Sn incorporation.^37^ The Ge–Ge LO mode in Ge_1–*x*_Sn_*x*_ has previously been shown to progressively shift toward a lower frequency with an increasing Sn concentration, due to the incorporation of Sn in the Ge lattice, altering the bond energy of the lattice.^8,47−49^ A much larger red shift in the frequency is observed for these Ge_1–*x*_Sn_*x*_ (0.1 ≤ *x* ≤ 0.35) nanowires compared to Ge_1–*x*_Sn_*x*_ nanowires with ∼9 atom % Sn incorporation.^37^ The observed difference in the peak position and bandwidth (11.4 cm^–1^ compared to 7.2 cm^–1^ for the cluster with larger Raman shift) for different Ge_1–*x*_Sn_*x*_ clusters could be due to the different Sn distribution in these clusters. Notably, a larger Raman shift, resulting from higher Sn incorporation, was observed for Ge_1–*x*_Sn_*x*_ nanowire clusters with thinner nanowire diameter distribution (38 nm for cluster II compared to 43 nm for cluster I) in the cluster (Figure S6, Supporting Information). However, the presence of different amounts of spherical aggregation in the clusters may also contribute toward the different Raman shift.

**Figure 3:**
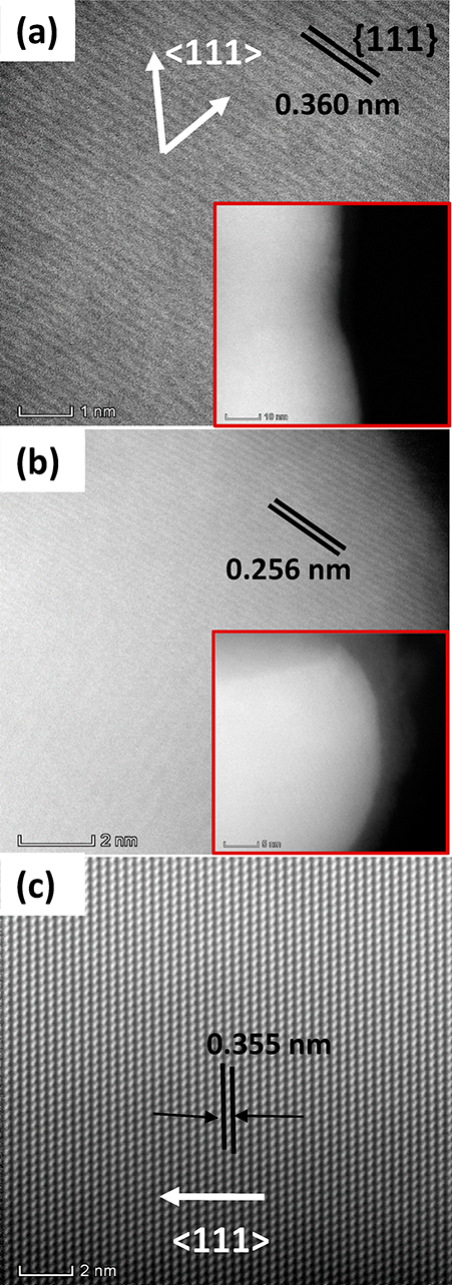
STEM analysis of Ge_1–*x*_Sn_*x*_ nanowires. (a) STEM image for Ge_1–*x*_Sn_*x*_ nanowire with high (∼20 atom %) Sn content. The low resolution of the image is associated with the deformation of the Ge_1–*x*_Sn_*x*_ nanowire by the electron beam. The inset shows low-resolution STEM of the corresponding nanowire. (b) STEM image of the spherical tip after the nanowire growth shows the formation of a predominantly Sn rich part at the tip. (c) High resolution STEM image of Ge_1–*x*_Sn_*x*_ nanowire with around 10 atom % Sn shows high crystal quality and ⟨111⟩ growth direction.

Determining the structural quality of the Ge_1–*x*_Sn_*x*_ nanowires is imperative as the high Sn content can result in the formation of crystal defects, such as twin boundaries or stacking faults, due to the large lattice mismatch between Ge and Sn. However, TEM/STEM analysis of the SCF grown Ge_1–*x*_Sn_*x*_ nanowires (especially for thinner nanowires where *x* > 0.20) proved exceedingly difficult, as the high voltage electron beam caused the nanowires to amorphize and recrystallize resulting in irreparable deformation (Figures S7 and S8 in the Supporting Information) of the Ge_1–*x*_Sn_*x*_ nanowires and rendering good quality high-resolution imaging unattainable. Nanowire deformed severely with the electron-beam exposure (Figure S8 in the Supporting Information shows the deformation with exposure time) to form polycrystalline segments in the GeSn crystal, with the formation of nanoclusters near the surface of the nanowires. Similar interplannar spacing (“*d*” value) for the deformed and nondeformed segment of the crystal suggests similar crystal structures for both segments. Sn incorporation in Ge_1–*x*_Sn_*x*_ nanorods (*x* = 0.17) has previously been demonstrated to segregate out at temperatures above 200 °C and even at temperature below 200 °C for Ge_1–*x*_Sn_*x*_ nanorods with 28 atom % Sn.^35^ Before electron beam deformation, the Ge_1–*x*_Sn_*x*_ nanowires showed no noticeable defects or twin boundaries and were single-crystalline as confirmed from high-resolution STEM observation (Figure 3a). The interface between the catalyst seed and nanowire body was examined by high resolution STEM and is depicted in Figure 3b. A bright contrasted seed region was clear in the image with no apparent tailing or segregation of Sn from the seed, further confirming the formation of a sharp junction at the interface as indicated from the EDX elemental maps in Figure 2b. Higher quality dark field STEM images were obtained for Ge_1–*x*_Sn_*x*_ nanowires with a relatively lower Sn content (*x* = 0.10–0.11). Figure 3c depicts the defect free, single crystalline nature of a Ge_1–*x*_Sn_*x*_ nanowire with a Sn content of 10 atom %. Fast Fourier transform (FFT) analysis of the nanowire aligned to the ⟨110⟩ zone axis (Figure 3c inset) revealed an interplanar spacing (*d*) of 0.355 nm (compared to 0.360 nm for a nanowire with relatively high Sn content), which is significantly larger than the *d* value for bulk diamond Ge crystal of 0.326 nm (JCPDS 04-0545). This increase in the *d* spacing is to be expected upon the incorporation of Sn into the Ge host lattice due to the difference in the lattice constants of Ge and Sn which can instigate a lattice expansion.^8^ The large *d* spacing observed, compared to GeSn thin film,^50^ could be due to the absence of any compressive strain in the free-standing nanowires. The nanowires predominantly displayed a ⟨111⟩ growth direction (Figure 3c).

**Figure 4:**
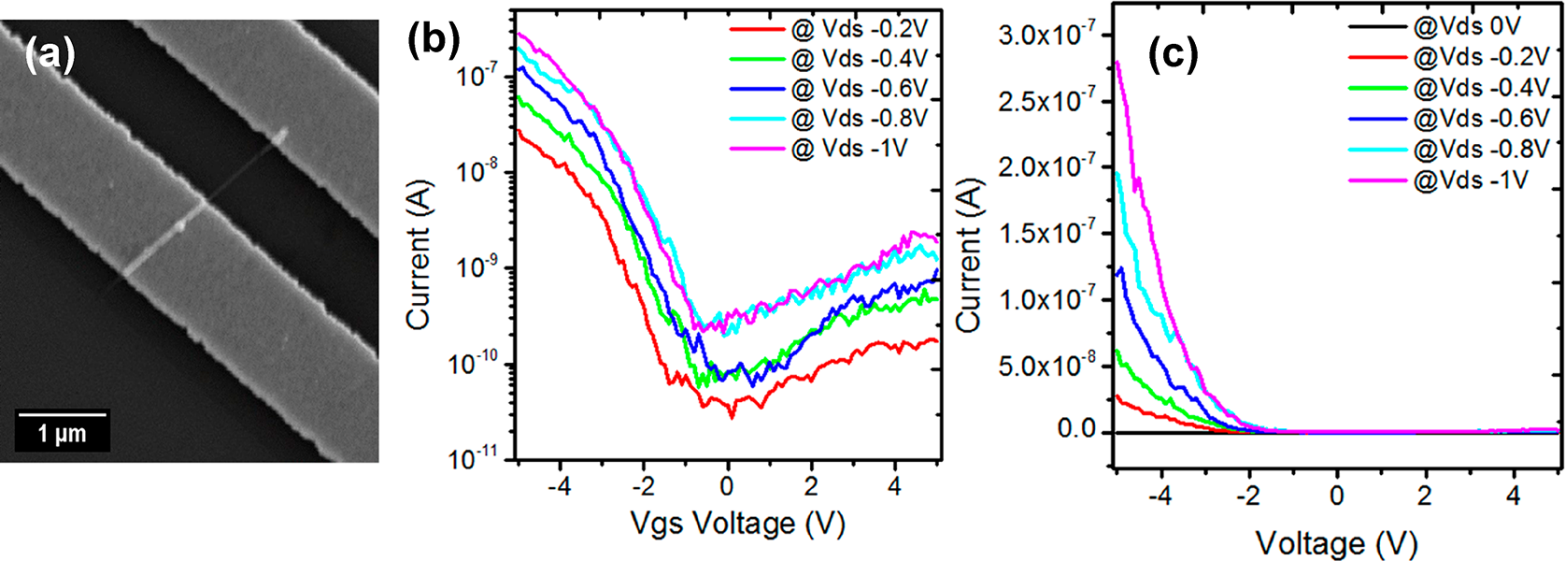
FET characteristics of Ge_1–*x*_Sn_*x*_ nanowires. (a) Illustrative image of the contacted Ge_0.9_Sn_0.1_ nanowire device. (b, c) Representative room temperature *I*_d_–*V*_gs_ characteristics with different *V*_ds_ values (−0.2 to −1 V).

The electrical field effect transistor (FET) characteristics of nominally undoped Ge_1–*x*_Sn_*x*_ nanowires (*x* = 0.10) were demonstrated by measuring the transfer characteristics (drain current (*I*_d_)–gate voltage (*V*_bg_)) as a function of source-drain voltage (*V*_ds_). Devices were fabricated by dropcasting a solution of nanowires in IPA onto highly doped Si with prepatterned UV contacts metallized by Ti–Au (5–25 nm). Electron-beam lithography was used to pattern contacts to individual wires which were etched in 10 % aqueous HCl for 5 min and metallized with 100 nm of Ni. Prior to electrical testing, nanowire devices were imaged by SEM to confirm the morphological quality of the devices formed and to determine the device geometry, e.g., channel length and nanowire diameter; a representative image is shown in Figure 4a. The electrical characteristics displayed in Figure 4 correspond to a device with a gate length of ∼780 nm and nanowire diameter of ∼45 nm. Nanowires of this particular diameter range had a Sn content of between 10 and 15 atom % (Figure 2c) and were deemed to have a lattice that is not deformed under electron beam, as observed from TEM analysis (Figure 3c). We did not generate any FET devices for Ge_1–*x*_Sn_*x*_ nanowires with very high Sn contents (>15 atom %), due to their structural instability (Figures S7 and S8 in the Supporting Information), which may also instigate possible deformation of the nanowire lattice under electrical bias. Additionally, the bandgap of Ge_1–*x*_Sn_*x*_ nanowires with a high Sn narrows, resulting in high off-state leakage in a FET device which is difficult to control.^51^

Unintentionally doped nanowires show linear *I*_ds_–*V*_ds_ characteristics between +1 V to −1 V, with *V*_bg_ = 0 (see Supporting Information, Figure S9). Contacts between the electrode and the nanowire were not ideal, as seen in the electrical features. This could result from an oxide layer at the contact and/or the relatively low dopant concentrations within the nanowires. The gate length was measured as the distance between the two metal contacts, as devices were back-gated. Transfer characteristic measurements were performed by sweeping the back-gate voltage between −5 to 5 V and setting the source-to-drain bias voltage as −0.2 to −1 V. The measurement range was carefully selected to prevent damaging the nanowires from high current densities and subsequent partial degradation. Figure 4b and Figure 4c show representative *I*_ds_–*V*_bg_ transfer characteristics of the Ge_1–*x*_Sn_*x*_ nanowires and highlight their ability to modulate the current even without intentional doping. Although the nanowires were nominally undoped, they displayed p-type semiconductor behavior in the sense that they operated at negative biases. For nominally undoped Ge_0.9_Sn_0.1_ this behavior is expected based on previous observations of p-type behavior in undoped VLS-grown Ge nanowires.^52^ The switching speed, or subthreshold slope (SS), in this case was 960 mV/dec, while on-current to off-current (*I*_ON_/*I*_OFF_) ratio was 2.25 × 10^2^. Top-gating and a gate-all-around device architecture would be necessary to reduce these SS values and increase *I*_ON_/*I*_OFF_. Considering the linear region of the current–voltage curves obtained, the carrier mobility (μ) was extracted from the transfer characteristics using eq 1:
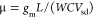
1where *L* and *W* are the nanowire gate length and channel width, respectively, *V*_sd_ is the bias between source and drain, and *C* is the capacitance for a back-gated nanowire device obtained using known values for ε, the dielectric constant of the underlying SiO_2_ layer, and nanowire diameter. The carrier mobility was determined to be 9.13 cm^2^/(V s) for Ge_1–*x*_Sn_*x*_ (*x* = 0.10). This mobility value is comparable to the carrier mobility obtained for the CVD grown Ge_1–*x*_Sn_*x*_ (*x* = 0.09) nanowires. However, the channel width for the FET devices from the CVD-grown nanowire was more than double (∼100 nm) compared to the FET devices fabricated from SCF-grown nanowires (see Table S1 in the Supporting Information for comparison of electrical parameters with CVD-grown Ge_1–*x*_Sn_*x*_ (*x* = 0.9) nanowires).^53^ Due to enhanced surface carrier scattering in a nanowire like structure, the mobility values are lower than those extracted in thick films which have minimal surface scattering effects.^54,55^

## Conclusion

We have reported the fabrication of Ge_1–*x*_Sn_*x*_ nanowires grown by a SCF approach. The introduction of high pressure resulted in a substantial increase in Sn in the nanowires, between 10 and 35 atom % with an average Sn content of 17.1 atom %. Despite the large Sn inclusion, the Ge_1–*x*_Sn_*x*_ nanowires produced did not display any apparent Sn segregation or clustering. Sn incorporation in the nanowires displayed a strong diameter dependence; small diameter nanowires contained higher amounts of Sn relative to their broader counterparts. Sn inclusion of up to *x* = 0.35 was achieved in Ge_1–*x*_Sn_*x*_ nanowires with diameters of approximately 20 nm. The diameter dependent inclusion of Sn is attributed to the growth kinetics in a diffusion limited VLS nanowire growth process. Thus, pressure can be an influencing factor to tune the amount of impurities in nanowires, especially in group IV nanowires. We believe this demonstration could positively influence nanowire growth research, especially doping and intentional and unintentional impurity incorporation in nanostructures. High resolution imaging of the nanowires formed revealed their single crystalline nature, with no apparent defects or twin boundaries. However, nanowires with a high Sn content (>15 atom %) displayed structural instability under a high voltage electron beam. Future effort on improving the morphological uniformity, uniformity in Sn content, and stability of high Sn content nanowire, by exploring new nanowire growth constraints, materials dimension, the use of epitaxial substrate, inclusion of third element such as Si in the alloy, etc. is required for the potential implementation of these materials in nanoelectronic, optoelectronic, and photonic devices. The electrical transfer characteristics of Ge_1–*x*_Sn_*x*_ (*x* = 0.10–0.11) nanowires obtained by forming back-gated FET devices suggest the potential of GeSn alloy nanowires in back-end-of-line integration schemes in nanoelectronic chip production. However, further effort and analysis on these nanowire system, regarding passivation, electrode formation, etc., are needed to improve the electrical performance.

## Supporting Information Available

Experimental description and details on the characterization techniques, diameter analysis of nanowires, EDX analysis of the GeSn nanowires, TEM analysis of high Sn content GeSn nanowires, *I*_d_–*V*_d_ curve for GeSn nanowire FET devices (PDF)
