# Supplementary material for: Stretching the Equilibrium Limit of Sn in Ge1–xSnx Nanowires: Implications for Field Effect Transistors
Source: ACS Appl Nano Mater. 2021 Feb 3;4(2):1048–56. doi: 10.1021/acsanm.0c02569 (PMC8153542; doi:10.1021/acsanm.0c02569)
Supplement: Supplementary file 1 — an0c02569_si_001.pdf [file an0c02569_si_001.pdf]

Supporting Information

Stretching the Equilibrium Limit of Sn in $Ge_{1-x}Sn_x$ Nanowires: Implications for Field Effect Transistors

Subhajit Biswas[1,2]*, Jessica Doherty[1,2], Emmanuele Galluccio[2], Hugh G. Manning[3], Michele Conroy[4], Ray Duffy[2], Ursel Bangert[4], John Boland[3] and Justin D. Holmes[1,2]

[1]School of Chemistry and Advanced Materials and Bioengineering Research (AMBER) Centre, University College Cork, Cork, T12 YN60, Ireland. [2]Tyndall National Institute, University College Cork, Cork, T12 R5CP, Ireland. [3]School of Chemistry and AMBER, Trinity College Dublin, Dublin 2, Ireland. [4] TEMUL, Department of Physics, Bernal Institute, University of Limerick, Limerick, V94 T9PX, Ireland.

Corresponding Author

Tel: +353 (0)21 490 5143; E-mail: s.biswas@ucc.ie

Experimental

For the catalysation of $Ge_{1-x}Sn_x$ nanowires in a three phase bottom-up growth dodecanethiol-stabilised, phase pure, $Au_{0.90}Ag_{0.10}$ alloy nanoparticles were used. Colloidal nanoparticles were synthesised by co-reducing a mixture of chloroauric acid ($HAuCl_4$) and silver nitrate ($AgNO_3$) in a chloroform/water biphasic solution.[1] These metal nanoparticles were deposited on to a Si(001) substrate with native oxide via spin coating and loaded into a stainless steel cell.

$Ge_{1-x}Sn_x$ nanowires were grown in 5 ml stainless steel cells (High Pressure Equipment Company) at a temperature of 405 °C. The handling of dry toluene, diphenylgermane (DPG) and allyltributylstannane (ATBS), as well as the filling of the reaction vessel and injection cell was carried out in a N_2 glove box under stringent precautions against water. The reaction cell and injection equipment were dried under vacuum at 180 ºC for 48 h and transferred into a glove box. 3 ml toluene was added into the 5 ml reaction cell and the assembly was heated to the desired temperature in a tube furnace for 2 h. After setting the pressure of the reaction cell to 21 MPa, the injection cell, filled with 20 ml of toluene/diphenylgermane/allyltributylstannane solution, was pressurized by an ISCO HPLC pump. The concentration of DPG in toluene was fixed at 5 µmol ml^{-1} whereas tin precursor concentrations were fixed at 0.75 µmol ml^{-1} for the incorporation of different amounts of Sn in the $Ge_{1-x}Sn_x$ nanowires. The precursor solution was injected at a rate of 0.025ml min^{-1} over a time period of 120 min under constant pressure conditions in a flow through reaction. Finally, the reaction cell was cooled to room temperature, depressurized and disassembled to access the growth substrate.

Characterization

Bottom-up grown $Ge_{1-x}Sn_x$ nanowires were imaged on a FEI Helios NanoLab 600i scanning electron microscope (SEM). All energy-dispersive X-ray (EDX) measurements were recorded in high-angle annular dark-field mode in the FEI Helios NanoLab 600i operating at 30 kV and 0.69 nA with an attached Oxford X-Max 80 detector. Error in the EDX measurements indicates a standard error of 0.5 at. %. TEM analysis was performed on a JEOL JEM-2100 operating at 200 kV in bright-field condition for imaging. EDX mapping was performed on a Titan Themis double-corrected and monochromated Transmission Electron Microscope at 300kV with a Bruker Super X detector. Software for imaging and EDS mapping is FEI Velox.

Field effect transistor (FET) devices were fabricated by drop-casting a solution of nanowires in IPA onto highly doped Si with pre-patterned UV contacts metallized by Ti-Au (5-25 nm). Electron-beam lithography was used to pattern contacts to individual wires which were etched in 10 % aqueous HCl for 5 min and metallized with 100 nm of Ni. Electrical measurements at room temperature were carried out with a Cascade semiautomatic prober station and an Agilent (HP) 4156C Parameter Analyser. To minimize the influence of the light the measurements were made in a dark ambient with a detected leakage current in the range of pico Amperes.

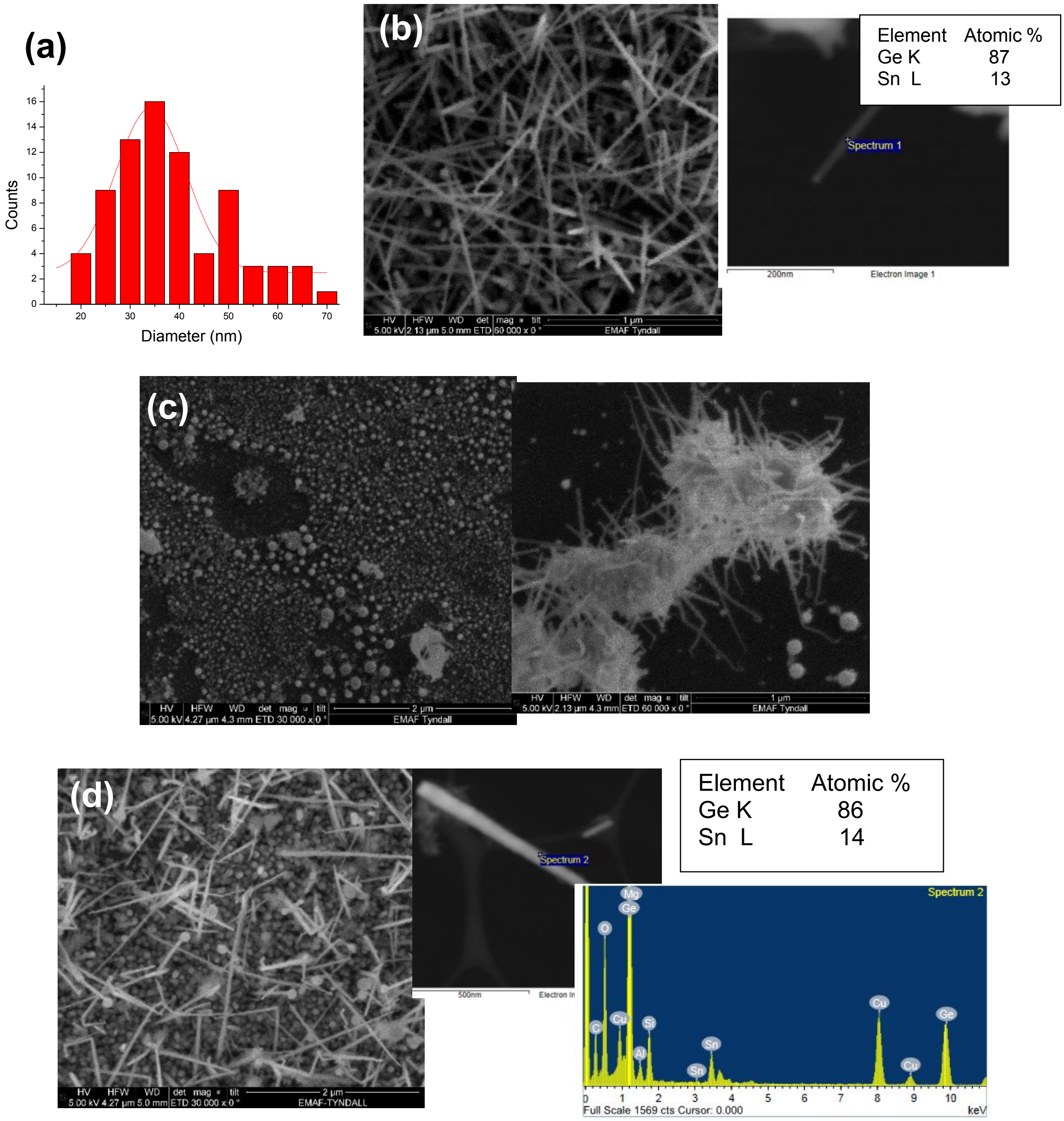

Figure S1: (a) Diameter distribution of GeSn nanowires. (b) SEM image and corresponding EDX analysis of $Ge_{1-x}Sn_x$ nanowires grown at 430 ºC, with other growth parameters similar to the standard growth parameters (21 MPs pressure, AuAg alloy catalysts, DPG and ATBS as Ge and Sn source). (c) SEM images show $Ge_{1-x}Sn_x$ nanowires (or lack of it) grown with Au catalyst. (d) SEM image and corresponding EDX analysis of the $Ge_{1-x}Sn_x$ nanowires grown with TET Sn precursor. Sample shows large amount of spherical segregation, tapered nanowires and nanowire with 8-15 at.% Sn.

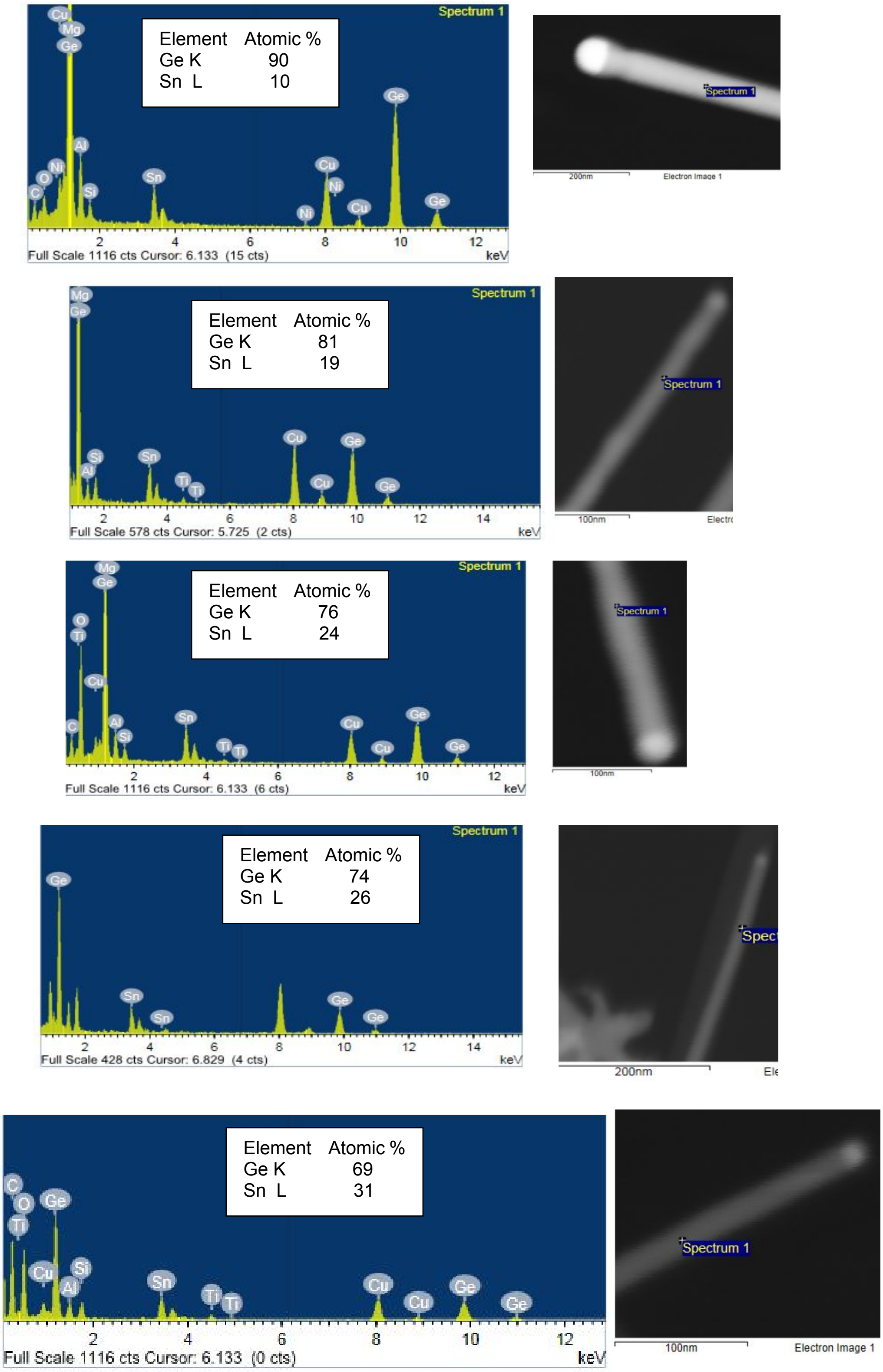

Figure S2: EDX elemental point analysis of $Ge_{1-x}Sn_x$ nanowires of different diameter shows increase in Sn inclusion with narrowing the diameter. EDX signals from other impurity elements (Si, Al, Ti etc.) are originating from the STEM detector and sample holder.

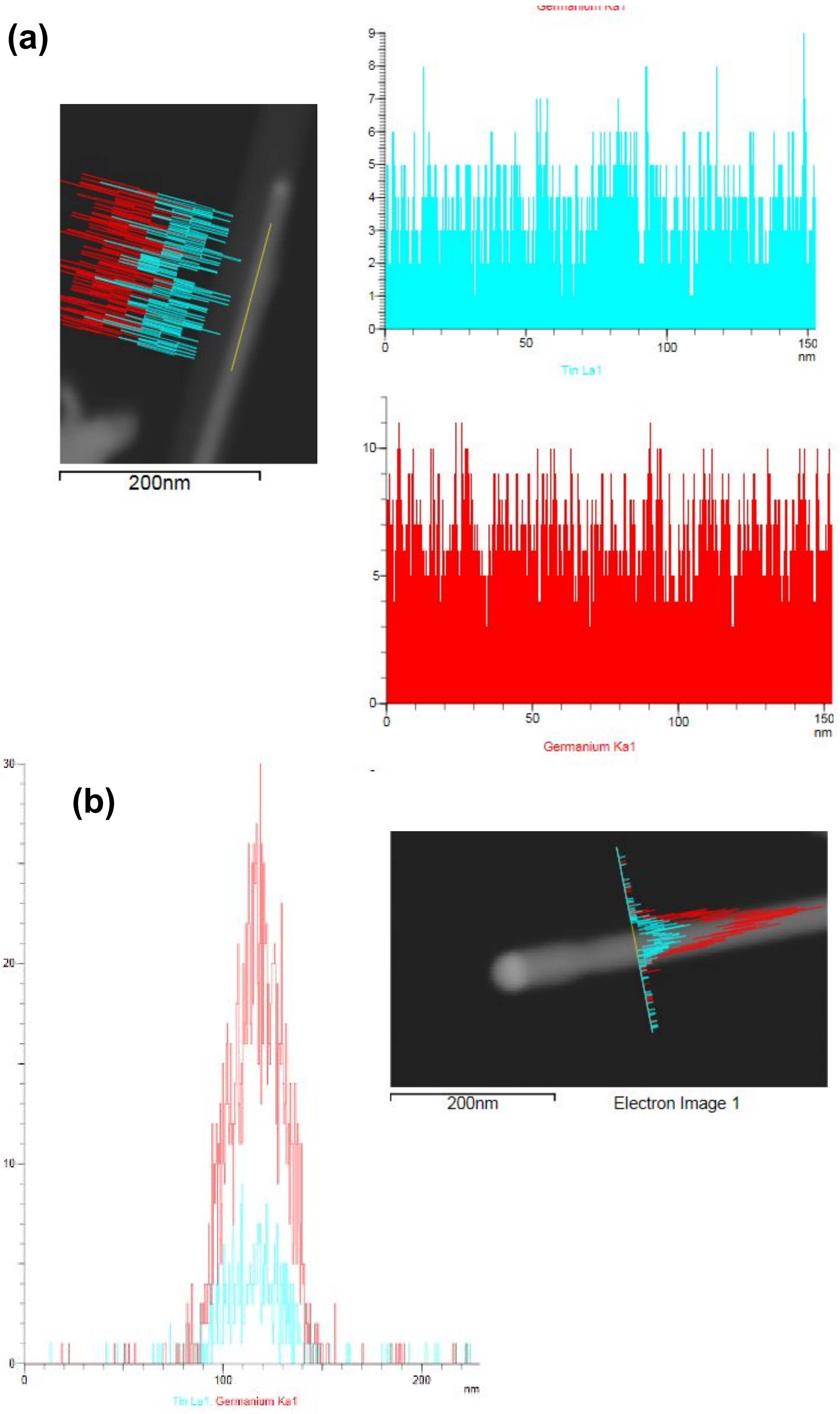

Figure S3: EDX line scan of a GeSn alloy nanowire with > 25 at.% Sn inclusion. (a) Axial line-scan (b) radial line-scan.

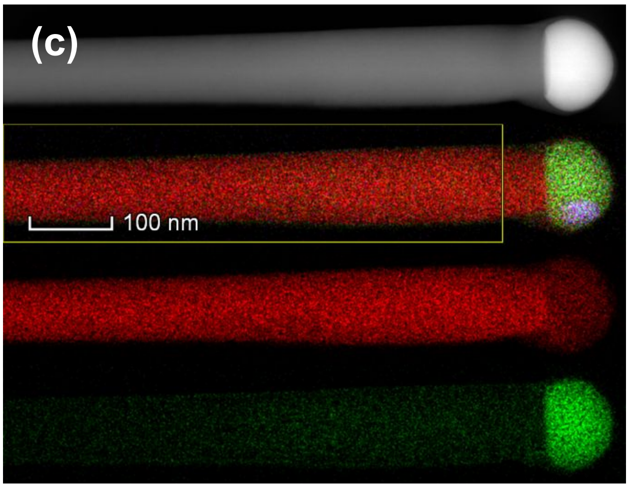

Figure S3 (c): EDX map of a relatively thicker GeSn nanowire.

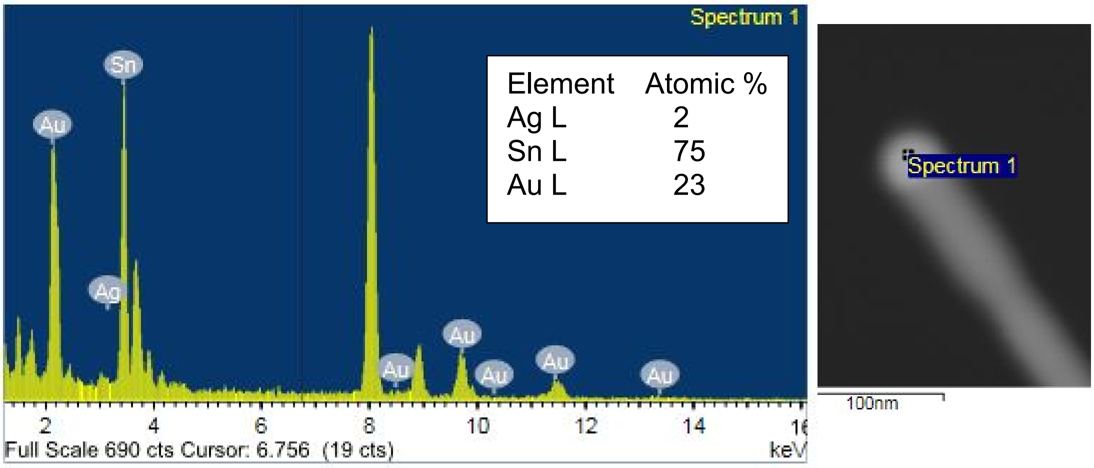

Figure S4: EDX elemental point analysis of the spherical tip of the $Ge_{1-x}Sn_x$ nanowires shows the formation of AuAgSn alloys with large Sn inclusion.

.

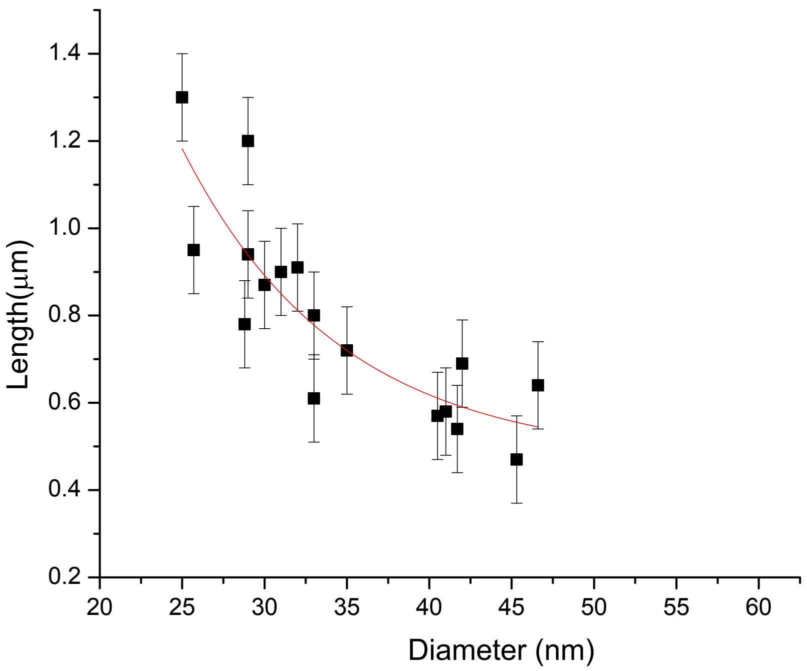

Figure S5: Nanowire diameter vs. length plot for $Ge_{1-x}Sn_x$ nanowires showing longer nanowire formation for smaller diameter.

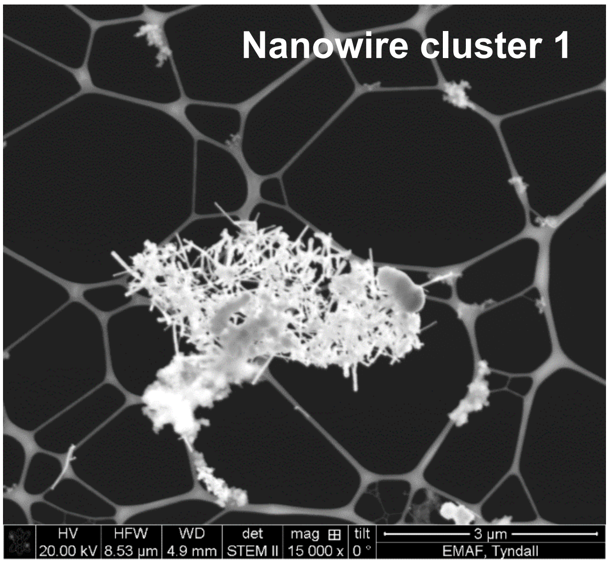

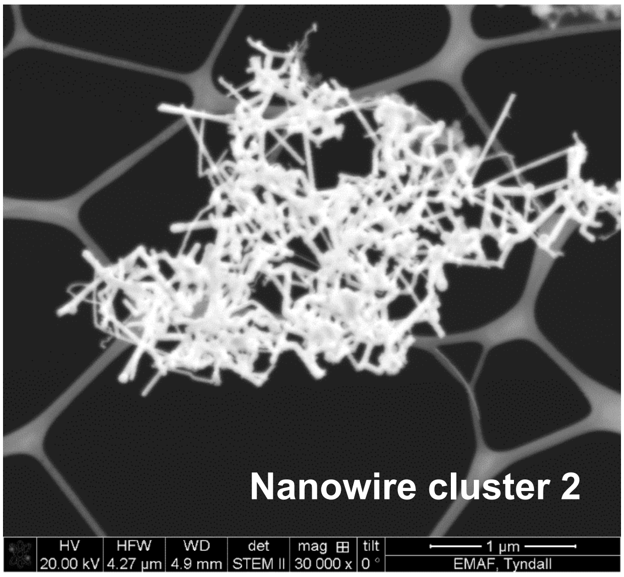

Figure S6: Dark-field STEM images showing the nanowire clusters from where the Raman measurements were recorded.

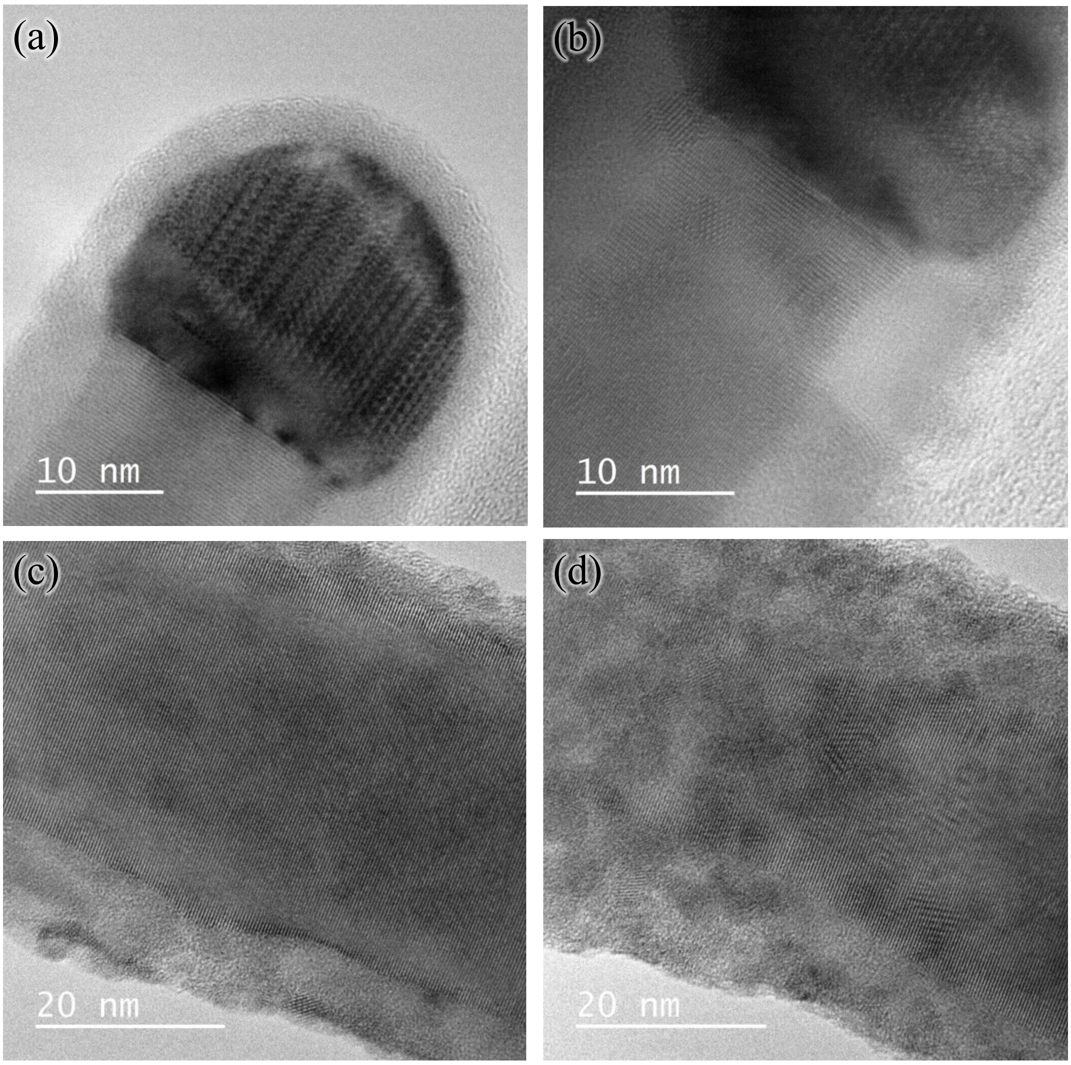

Figure S7: TEM analysis of $Ge_{1-x}Sn_x$ nanowires. TEM images taken before (a) and after (b) deformation of the $Ge_{1-x}Sn_x$ nanowire by the electron beam at the seed/body interface and before (c) and after (d) deformation in the bulk of the $Ge_{1-x}Sn_x$ nanowire. As the deformation of the crystal started instantly after the e-beam exposure, amorphization and nanoparticle formation can be seen even in the TEM image in part (c).

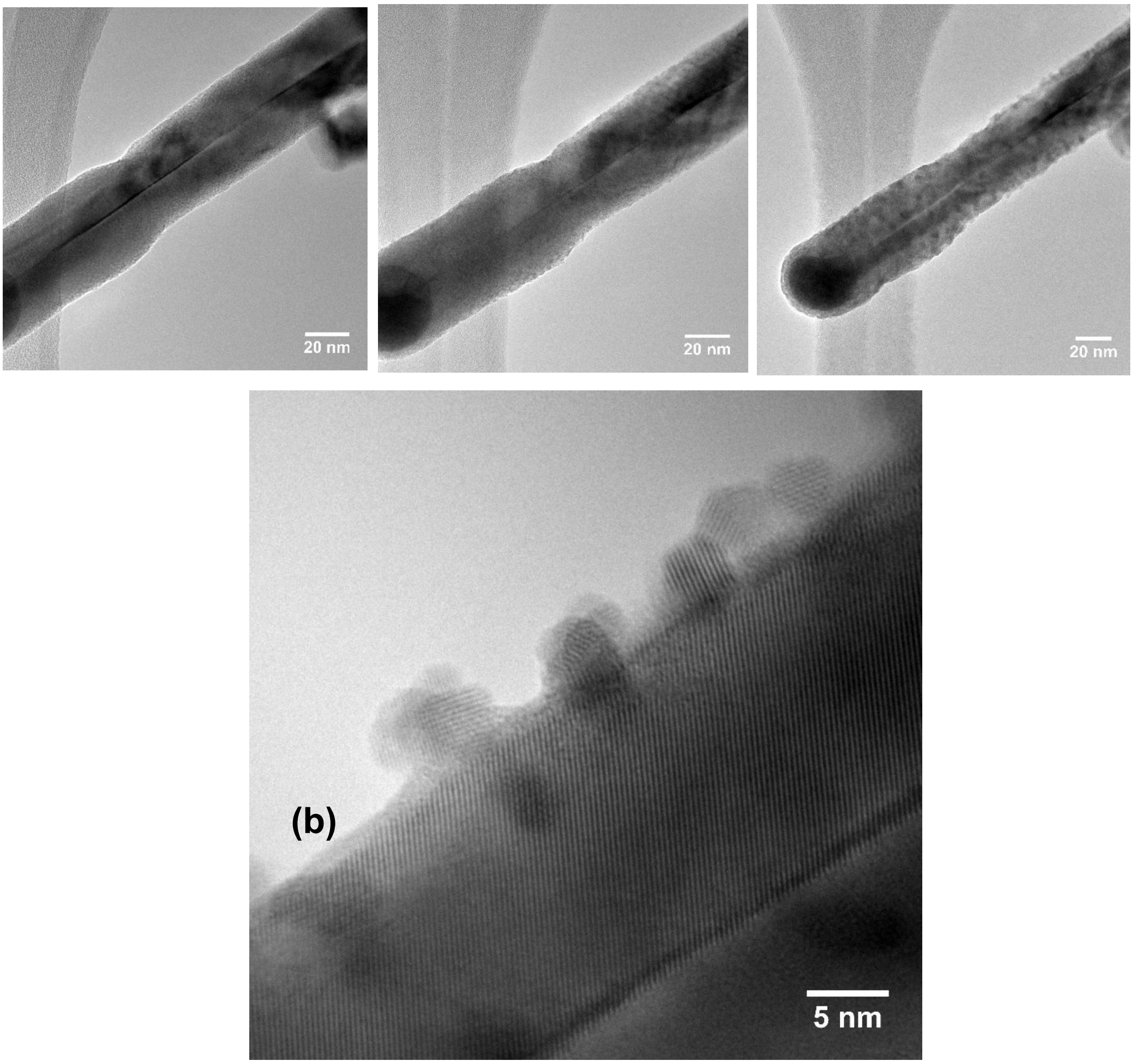

Figure S8: (a) TEM images of $Ge_{1-x}Sn_x$ nanowires show deformation with time (with 1 min interval). (b) No apparent change in the crystal lattice was observed for the deformed and non-deformed area of the nanowire.

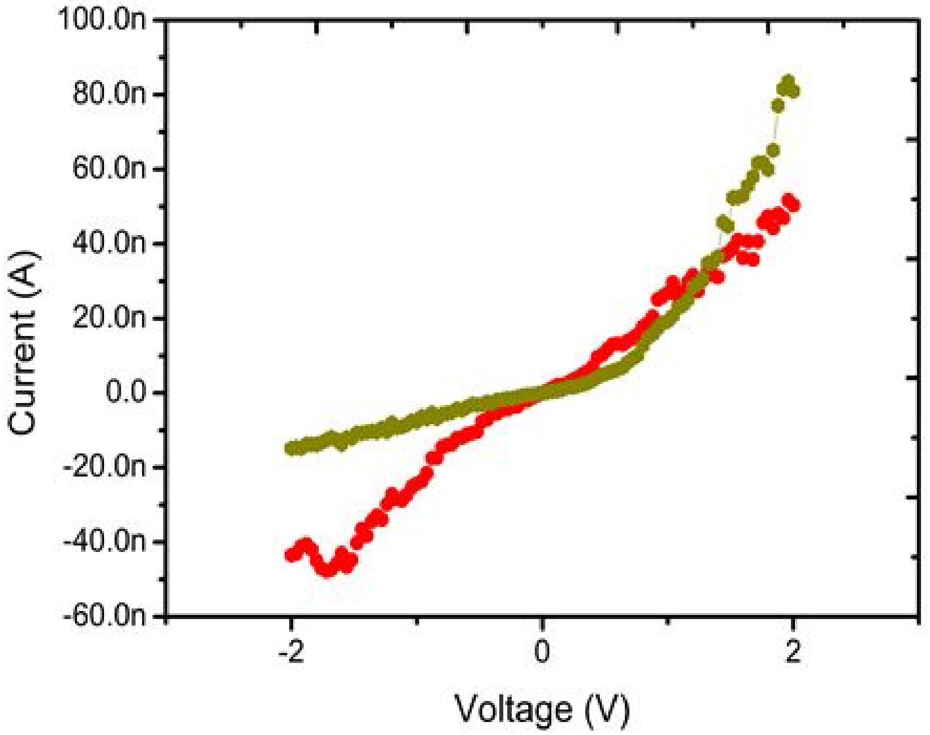

Figure S9: : Representative I_d-V_d curve for is $Ge_{0.90}Sn_{0.10}$ nanowire devices. To perform the transfer characteristics (Figure 4 in the main manuscript) nanowire FET device with red curve was selected. Transfer characteristics were performed in the linear region of V_{ds} (-1V to 1V).

Table S1: Comparison of the transistor parameters between CVD grown $Ge_{1-x}Sn_x$ (x= 0.9)[2] and nanowire and SCF grown $Ge_{1-x}Sn_x$ (x= 0.9) nanowires. Fabrication parameters are similar except the surface passivation method: HCl passivation for the SCF-grown nanowires and buffered oxide etch for CVD-grown nanowires.

**Year**	**CVD grown nanowires**	**2020 this work**
**Sn%**	9	10
**Proc.T.**	<440°C	<405°C
**Dev. Struc**	NWs (bot. gate)	NWs (bot. gate)
**Channel diameter**	100 nm	45 nm
**Peak μ**	μ=14.8 (cm^2/Vs)	μ=9.13 ($cm^2/Vs)$
**I_{on}/I_{off} ratio**	7.8×10^2	2.25×10^2
**SS (mV/dec)**	1525	960

References:

[1] He, S. T.; Xie, S. S.; Yao, J. N.; Gao, H. J.; Pang, S. J.; *Applied Physics Letters* **2002,** *81*, 150.

[2] Galluccio, E.; Doherty, J.; Biswas, S.; Holmes, J. D.; Duffy, R. Field-Effect Transistor Figures of Merit for Vapor–Liquid–Solid-Grown Ge 1-x Sn x (x = 0.03–0.09) Nanowire Devices . *ACS Appl. Electron. Mater.* **2020**, *2* (5), 1226–1234.
